# Follow-up in Patients With Non-invasive Prenatal Screening Failures: A Reflection on the Choice of Further Prenatal Diagnosis

**DOI:** 10.3389/fgene.2021.666648

**Published:** 2021-05-19

**Authors:** Sha Liu, Hongqian Liu, Jianlong Liu, Ting Bai, Xiaosha Jing, Tianyu Xia, Cechuan Deng, Yunyun Liu, Jing Cheng, Xiang Wei, Lingling Xing, Yuan Luo, Quanfang Zhou, Qian Zhu

**Affiliations:** ^1^Department of Obstetrics and Gynecology, West China Second University Hospital, Sichuan University, Chengdu, China; ^2^Key Laboratory of Birth Defects and Related Diseases of Women and Children, Ministry of Education, Sichuan University, Chengdu, China

**Keywords:** non-invasive prenatal screening, cell-free fetal DNA, test failure, no-call, prenatal diagnosis

## Abstract

**Background:**

Our aim was to provide a theoretical basis for clinicians to conduct genetic counseling and choose further prenatal diagnosis methods for pregnant women who failed non-invasive prenatal screening (NIPS).

**Methods:**

A retrospective analysis was performed on pregnant women who had failed NIPS tests.

**Results:**

Among the 123,291 samples, 394 pregnant women did not obtain valid results due to test failures. A total of 378 pregnant women were available for follow-up, while 16 patients were lost to follow-up. Of these 378, 135 pregnant women chose further prenatal diagnosis through amniocentesis, and one case of dysplasia was recalled for postpartum chromosome testing. The incidence rate of congenital chromosomal abnormalities in those who failed the NIPS was 3.97% (15/378), which was higher than that of the chromosomal abnormalities in the common population (1.8%). Among the pregnant women who received prenatal diagnosis, the positive rates of chromosomal abnormalities in the chromosomal microarray analysis/copy number variation sequencing (CMA/CNV-seq) group and in the karyotyping group were 15.28 and 4.76%, respectively.

**Conclusion:**

Prenatal diagnosis should be strongly recommended in posttest genetic counseling for pregnant women with NIPS failures. Further, high-resolution detection methods should be recommended for additional prenatal diagnoses.

## Introduction

In recent years, non-invasive prenatal screening (NIPS) using cell-free fetal DNA (cffDNA) in the peripheral blood of pregnant women has been widely used because of its high specificity, sensitivity, and non-invasive characteristics for screening common fetal chromosomal aneuploidies (trisomy 21, trisomy 18, and trisomy 13). Gil et al. (2017) conducted a meta-analysis of 35 relevant studies and found that screening approximately 220,000 NIPS test samples collected in 2017 in singleton pregnancies could detect up to 99% of trisomy 21, 98% of trisomy 18, and 99% of trisomy 13 at a combined false-positive rate (FPR) of 0.13%.

However, NIPS detection failure always exists in clinical practice. A statement from the Chromosome Abnormality Screening Committee on behalf of the Board of the International Society for Prenatal Diagnosis (ISPD) declared that the NIPS detection failure rate is approximately 1.9–6.4% in some large laboratory reports (Benn et al., 2015). Similarly, the detection failure rates were reported by different sequencing platforms (Verinata, LifeCodexx, Sequenom, BGI, Ariosa, Natera) range from 1.6 to 6.4% (Cuckle, 2017). The American College of Obstetricians and Gynecologists (ACOG) and the Society of Maternal-Fetal Medicine (SMFM) have proposed that the “no-call” (i.e., test failure) group is at an increased risk of chromosomal aneuploidy in the test NIPS consultations, suggesting that these patients should receive genetic counseling and further diagnostic tests (Committee on Genetics, and The Society for Maternal-Fetal Medicine, 2015; Committee on Practice Bulletins-Obstetrics, Committee on Genetics, and The Society for Maternal-Fetal Medicine, 2016b). In a study by Norton et al. (2015), the detection rate of chromosome aneuploidy was 0.36% (57/15841) in the successful NIPS group and 2.25% (11/488) in the failed NIPS group. In a study by Revello et al. (2016), the detection rate of chromosome aneuploidy was 2.1% (218/10382) in the successful NIPS group and 2.53% (8/316) in the failed NIPS group. In actual clinical genetic counseling, doctors recommend invasive prenatal diagnosis for pregnant women who fail NIPS. Regarding prenatal diagnosis, the available methods are karyotyping, chromosomal microarray analysis (CMA), copy number variation sequencing (CNV-seq), fluorescence *in situ* hybridization (FISH), and quantitative fluorescence polymerase chain reaction (QF-PCR). In previous studies of NIPS failure, almost all the prenatal diagnostic tests were karyotype analysis or FISH (Pergament et al., 2014; Norton et al., 2015; Palomaki et al., 2015; Revello et al., 2016; Suzumori et al., 2016). With the wide clinical application of high-resolution detection methods such as CMA, it is possible to find pathogenic copy number variations beyond the detection range of karyotype analysis (Hay et al., 2018). However, no studies have shown the results of using high-resolution methods in the prenatal diagnosis of patients with NIPS failure. Thus, we performed a large sample size retrospective analysis of NIPS test failure cases. By analyzing the prenatal diagnosis methods, the results of NIPS test failure cases, and the associated pregnancy outcomes, the incidence of chromosomal aberrations in NIPS test failure cases was obtained. Our results will provide more evidence for the clinical selection of prenatal diagnosis in patients with NIPS failure.

## Materials and Methods

### Materials

This retrospective study was conducted from April 2015 to June 2019 in pregnant women undergoing prenatal screening and diagnosis at West China Second University Hospital of Sichuan University, Chengdu, Sichuan Province, China. A total of 394 pregnant women who had failed the NIPS test during this time period participated in our study. All participants who performed the NIPS test received professional pretest counseling and were fully aware of the scope of application, target diseases, limitations, and possible inability to obtain results due to low fetal DNA concentration or other reasons. The study was approved by the Institutional Ethics Committee of Sichuan University, and all participants signed written informed consent prior to testing. We confirmed that the research was performed in accordance with relevant clinical technical specifications.

### Non-invasive Prenatal Screening

According to standard operating procedures, maternal peripheral blood (8–10 mL) was collected from all participants using Cell-free BCT Tubes (Streck, Omaha, NE, United States). The collected blood samples were centrifuged twice within 72 h using an Eppendorf 5810R and 5430R centrifuge (Eppendorf, Hamburg, Germany). The upper plasma was collected after centrifugation. cfDNA was isolated from 1200 μL plasma using a DNA extraction kit (Hangzhou Berry Gene Diagnostic Technology Co., Ltd., Hangzhou, China). The remaining plasma was stored at −70°C. The cfDNA concentration was measured using a Qubit 3.0 and ExKubit dsDNA HS test kit (ExCell Biotech Co., Ltd., China). The normal cfDNA concentrations should be ≥0.05 and <0.6 ng/μL. If the concentration was out of this range, the DNA was re-extracted. If the concentration was unqualified again, it was regarded as a test failure. A non-invasive prenatal test library prep kit (Reversile Terminator Sequencing) (Hangzhou Berry Gene Diagnostic Technology Co., Ltd., Hangzhou, China) was used for library construction. In this process, an identifiable index sequence was added to each sample library. The quality and concentration of the libraries were detected using a KAPA SYBERFAST qPCR kit (KAPA Biosystems, Wilmington, MA, United States). The libraries were sequenced using a NextSeq CN500 high-throughput sequencing kit (Illumina) on NextSeq CN500 platform (Illumina). Each sample generated approximately 5 million raw data with 36-bp reads. Approximately 3 million reads were uniquely mapped to the hg19 genomic sequence. The z-values (normal range, –3 < Z < 3) of 24 chromosomes were further calculated from the normalized sequencing data after the removal of abnormal GC regions and low-coverage regions. Sequencing reactions that did not yield definitive results (sequencing failure) or fetal fraction <4% twice were regarded as test failures.

### Invasive Prenatal Diagnosis

All pregnant women who failed the NIPS test received a posttest clinical consultation provided by a qualified clinical geneticist, where they could choose whether to perform invasive prenatal diagnosis. The diagnostic methods available for pregnant women included CNV-seq, CMA, and karyotyping. The method used depended on the option the pregnant woman chose after clinician performed genetic counseling. Regardless of the chosen method, the laboratory analyzed each sample with QF-PCR to quickly determine the chromosome 21/18/13 and sex chromosome aneuploidy and assess maternal blood cell contamination (MCC). When the results of the two detection methods conflicted, additional FISH analysis was performed for verification. Only some of the pregnant women in this study chose amniocentesis for subsequent detection.

#### Copy Number Variation Sequencing

Genomic DNA was extracted from amniotic fluid cells using a DNeasy Blood and Tissue Kit (Qiagen GmbH, Hilden, Germany). DNA libraries were constructed. DNA libraries were sequenced using a NextSeq CN500 platform (Illumina). CNVs were classified by querying public databases, including DGV,^1^ DECIPHER,^2^ OMIM,^3^ and PubMed.^4^

#### Chromosomal Microarray Analysis

It was performed using a CGX v2 Oligo aCGH Kit (Agilent Technologies, Santa Clara, CA, United States, and Perkin Elmer, Turku, Finland). CMA single-nucleotide polymorphisms were analyzed using an Affymetrix CytoScan 750K Array (Affymetrix, Santa Clara, CA, United States). The clinical significance of the detected CNVs was systematically evaluated (Hu et al., 2019).

#### Karyotyping

Amniotic fluid cells were cultured in AmnioMAX-II (Gibco, Carlsbad, CA, United States) medium. Each specimen was analyzed by g-banding at a resolution of above 400 bands on average (minimum 320 bands required).

#### Quantitative Fluorescence Polymerase Chain Reaction

Quantitative fluorescence polymerase chain reaction was performed on chromosomes 13, 18, 21, X, and Y of all prenatal diagnostic samples. MCC was also assessed. Several loci of chromosomes 13, 18, 21, X, and Y were detected with probes labeled with short-tandem repeat (STR). Alleles were screened and labeled using genetic mapping software (Applied Biosystems, Foster City, CA, United States). Normal allelic dose ratios were between 0.8 and 1.4. When QF-PCR results were not available or the marker information of a chromosome was incomplete, FISH was used for further analysis (GP Medical, Beijing, China).

#### FISH

Fluorescence *in situ* hybridization analysis was performed using the Prenatal Chromosome Testing Kit (Fluorescence *in situ* hybridization) (GP Medical, Beijing, China) according to the manufacturer’s protocol.^5^ After the fluorescent-labeled DNA probe was hybridized with the sample DNA, the fluorescent signal was detected using a fluorescent microscope to assess the chromosome aneuploidy.

### Statistical Analysis

Statistical Product and Service Solutions (SPSS) version 21.0 (SPSS Inc., Chicago, IL, United States) was used for data analyses. Data are presented as means ± SD. ANOVA, and independent sample *t*-tests were used to compare different groups. *P* < 0.05 was considered statistically significant.

## Results

Between April 2015 and June 2019, a total of 123,291 pregnant women underwent NIPS at the West China Second Hospital of Sichuan University; almost all of them were Chinese. Of these, 122,897 women received formal clinical reports, while 394 failed to obtain valid results due to test failures. Patients who entered the NIPS test stage and failed the final test were included in this study. All the participants provided informed consent to participate in the study.

### Basic Information on NIPS Test Failure

A total of 394 cases were classified as test failure (or obtained a no-call result), and the overall test failure rate was 0.32% (394/123,291). The average maternal age of patients who failed the NIPS test was 29.46 ± 4.80 years, the average gestational age was 17.78 ± 2.93 weeks, and the average body mass index (BMI) was 25.00 ± 3.77 kg/m^2^. The causes of test failure were summarized into three categories: low fetal fraction (fetal fraction < 4%), sequencing that did not yield definitive results, i.e., sequencing failure, and a DNA concentration that exceeded the quality control standard after extraction. Table 1 summarizes the characteristics of patients with different reasons for test failure. A low fetal fraction was the main reason for the failure of NIPS testing (79.44%) (Table 1).

**TABLE 1:** The basic characteristics of NIPS test failure cases.

**Reason for test failure**	**n (%)**	**Gestational age (weeks)**	**Maternal age (years)**	**BMI (kg/m^2^)**
Fetal fraction < 4%	313 (79.44)	17.40 ± 2.27^A^	29.48 ± 4.79	25.66 ± 3.67^D^
Sequencing failure	16 (4.06)	19.44 ± 3.03^B^	29.12 ± 4.15	22.41 ± 2.58 ^E^
DNA concentration outside of laboratory quality control	65 (16.50)	19.20 ± 4.72^*C*^	29.45 ± 5.08	22.45 ± 3.06^F^
Total	394 (100.00)	17.78 ± 2.93	29.46 ± 4.80	25.00 ± 3.77

*BMI (body mass index) = weight (kg)/height (m)^2^. Independent-sample t-tests were used to compare gestational age and BMI in different groups, A vs B: *p* = 0.001, A vs C: *p* = 0.004, D vs E: *p* = 0.001, D vs F: *p* < 0.001.*

### Prenatal Examinations and Pregnancy Outcomes of NIPS Test Failure Cases

In a further follow-up of the 394 test failures, 16 cases were lost to follow-up, and 378 obtained follow-up results. The first follow-up was conducted 3–6 months after the NIPS. If the pregnancy outcome was not obtained in the first follow-up, a second follow-up was conducted half a year after delivery. The mean follow-up period was 267 days after delivery (or termination of pregnancy). One hundred and thirty-five cases (35.71%) chose invasive prenatal diagnosis, while 243 cases (64.29%) refused to undergo invasive prenatal diagnosis after genetic counseling. Fourteen cases (14/378, 3.7%) were identified with congenital chromosomal abnormalities in prenatal examinations (nine cases chose to continue the pregnancy, whereas five chose to terminate it; the specific pregnancy outcomes are shown in Table 2, case numbers P1–P14). In the 121 normal chromosome cases and 243 cases that rejected prenatal diagnosis, 38 had abnormal pregnancy outcomes (among the 38 cases, five cases had chromosome examination results, with case numbers S1, S2, and S4, and two cases had abortion tissue examinations, while the chromosome results of the remaining cases were unknown). Tables 3, 4 provide a detailed classification and summary of the abovementioned prenatal examinations and pregnancy outcomes.

**TABLE 2:** All chromosome abnormalities detected in 378 NIPS test failures.

**Case number**	**Gestational age (weeks)**	**Maternal age (years)**	**BMI (kg/m^2^)**	**Number of fetus**	**Detection method**	**Chromosomal abnormality**	**Pathogenicity rating**	**Follow-up outcomes**
P1	19	29	30.84	Single pregnancy	CNV-seq	seq[GRCh37]del(9)(q33.1)(0.84 Mb)	VUS	Continued pregnancy and gave birth
P2	19	27	20.20	Single pregnancy	CNV-seq	seq[GRCh37]del(8)(q21.3q22.1) (1.14 Mb)	VUS	Continued pregnancy and gave birth
P3	19	33	24.03	Single pregnancy	CNV-seq	seq[GRCh37]del(22)(q11.21q11.21) (2.57Mb)	P	Continue pregnancy and gave birth, growth retardation
P4	17	33	21.37	Single pregnancy	CNV-seq	seq[GRCh37]dup(8)(q24.11q24.11)(0.64 Mb)	VUS	Continued pregnancy and gave birth
P5	19	25	22.94	Single pregnancy	CNV-seq	seq[GRCh37]del(22)(q11.22q11.23)(0.66 Mb)	LP	Continued pregnancy and gave birth
P6	18	21	19.05	Single pregnancy	CNV-seq	seq[GRCh37]dup(11)(p15.5p15.4)(9.06 Mb), seq[GRCh37]del(10)(q26.2q26.3)(5.08 Mb), seq[GRCh37]dup(8)(q21.12q21.12)(0.62 Mb)	LP	Termination of pregnancy
P7	16	33	28.23	Single pregnancy	CNV-seq	seq[GRCh37]del(22)(q11.21q11.23)(2.62 Mb)	P	Termination of pregnancy
P8	18	34	25.91	Twin pregnancy	CNV-seq	fetus1:seq[GRCh37]del(10)(q26.3q26.3)(0.88 Mb) fetus2:seq(1-22) × 2,(XN) × 1	VUS	Continued pregnancy and gave birth
P9	18	35	21.60	Twin pregnancy	CNV-seq	fetus1:seq[GRCh37]del(11)(q14.2q22.1)(18.62 Mb) fetus2:seq(1-22) × 2,(XN) × 1	P	Selective termination of the affected fetus
P10	20	28	22.03	Single pregnancy	CNV-seq^a^	Mosaic XX/XY	P	Termination of pregnancy
P11	20	33	22.31	Single pregnancy	CNV-seq	seq[GRCh37]dup(Y)(p11.31q11.221)(15.61 Mb), seq[GRCh37]del(Y)(q11.221q11.23)(10.54 Mb)	P	Termination of pregnancy
P12	17	28	22.95	Single pregnancy	Karyotyping	47,XYY		Continued pregnancy and gave birth
P13	16	36	28.00	Single pregnancy	Karyotyping	46,XN,t(1;22) (q11;q11.2)		Continued pregnancy and gave birth
P14	19	30	25.00	Single pregnancy	Karyotyping	mos 45,X,9qh + [18]/46,XY,9qh + [32]		Continued pregnancy and gave birth
S4^*b*^	18	36	21.83	Single pregnancy	WES	*GATAD2B*:NM_020699.4:exon7:c.1178G > A:p.G393D	P	Comprehensive developmental delay and many other abnormalities (see Table 4)

*P, pathogenic; LP, likely pathogenic; VUS, variant of uncertain significance; XN, XX, or XY. Pathogenicity was rated only for detected CNVs or SNVs in the table. ^*a*^ The CNV-seq and QF-PCR results of this case were inconsistent. CNV-seq did not indicate abnormalities, but QF-PCR indicated abnormalities in the signal of sex chromosome sites. Additional FISH was performed, which suggested mosaic XX/XY. ^*b*^Case S4 was diagnosed after delivery.*

**TABLE 3:** Prenatal diagnosis and pregnancy outcomes of 378 NIPS test failures (excluding lost-to-follow-up cases).

**Characteristic**	**n**	**Proportion (%)**	**Remarks**
Performed invasive prenatal diagnosis	135	35.71	
Abnormal chromosomal results	14	3.70	
Normal chromosomal results	121	32.01	
Normal pregnancy outcomes	116	30.69	
Abnormal pregnancy outcomes	5	1.32	
Abnormal development after birth	2	0.53	See Table 4. Case numbers S1, S2
Premature delivery	1	0.26	Died after giving birth prematurely at 28 week
Miscarriage	2	0.53	No abortion tissue examination was performed
Rejected invasive prenatal diagnosis	243	64.29	
Normal pregnancy outcomes	210	55.56	
Abnormal pregnancy outcomes	33	8.73	
Abnormal development after birth	3	0.79	See Table 4. Case numbers S4, S5, and S6
Premature delivery	2	0.53	One subject died 2 months after birth from pulmonary dysplasia, one subject died after preterm birth at 27 + 2 weeks
Miscarriage	13	3.44	Only two cases underwent abortion tissue examinations, and the results were normal. The reasons for the rest cases are unknown
Induced labor	15	3.97	Five cases had prenatal ultrasound malformation or other abnormalities, two cases had stillbirth, three cases had serious pregnancy complications, and five cases had unknown causes

**TABLE 4:** Overview of cases of postnatal dysplasia.

**Case number**	**Signs of dysplasia**	**Prenatal chromosome examination results**	**Postpartum chromosome examination results**
S1	Dislocation of hip joint, difficulty crawling	Karyotyping: normal	Unwilling to undergo other higher resolution detection
S2	Genital abnormality	Karyotyping: normal	Unwilling to undergo other higher resolution detection
S3	Growth retardation	CNV-seq:(pCNV) del22q11.21q11.21 (2.57 Mb)	No additional detection was required
S4^*a*^	Comprehensive developmental delay, hypoxic ischemic brain injury, visual impairment, hearing impairment, secondary epilepsy, cryptorchidism	Not tested	WES: (Pathogenic) *GATAD2B*:NM_020699.4:exon7:c.1178G > A:p.G393D
S5	Cryptorchidism	Not tested	Unwilling to undergo other higher resolution detection
S6	One of the twins had no left auricle, and hearing was affected	Not tested	Unwilling to undergo other higher resolution detection

*pCNV, pathogenic copy number variation; WES, whole exome sequencing. ^*a*^Case S4 was diagnosed after delivery.*

### Invasive Prenatal Diagnosis and Chromosomal Abnormalities Were Detected

A total of 135 cases were selected for invasive prenatal diagnosis. All amniotic fluid samples containing fetal genetic material were obtained through amniocentesis. No fetal loss or other puncture complications occurred due to amniocentesis. The testing methods available for pregnant women included karyotyping, CMA, and CNV-seq.

There were 14 samples that suggested chromosomal abnormalities (Table 2). The participants were divided into two groups according to the resolution of the prenatal diagnosis method: karyotyping or CMA/CNV-seq (Table 5). In the karyotyping group (63 samples), one case of balanced translocation and two cases of sex chromosome abnormalities were detected. In the CMA/CNV-seq group (72 samples), one case of sex chromosome abnormality and 10 cases of copy number variations were detected. The overall detection rate of the CMA/CNV-seq group (15.28%) was higher than that of the karyotyping group (4.76%). The specific chromosomal abnormalities are shown in Table 2, which lists all 15 cases of chromosome abnormalities (14 cases were detected in prenatal diagnosis and 1 in postnatal examination) detected in 378 NIPS test failures, including abnormal types and pregnancy outcomes. These samples failed because of a low fetal fraction. Ten cases analyzed with CNV-seq showed CNV, and one case of whole exome sequencing (WES) showed single-nucleotide variation (SNV). The pathogenicity ratings of CNVs and SNV are shown in Table 2. In one case, the CNV-seq and QF-PCR results were inconsistent. The CNV-seq results for this sample did not indicate any abnormalities, whereas the QF-PCR results indicated abnormalities in the sex chromosome sites. Due to the inconsistent results, the sample was also analyzed with FISH. This analysis suggested a mosaic abnormal sex chromosome XX/XY: the X and Y probes detected one signal each in three cells, while the X probe detected two signals and the Y probe detected no signal in 97 cells. Three cases were indicated as chromosome aneuploidy (47, XYY), balanced translocation, and abnormal mosaicism by karyotyping.

**TABLE 5:** Selection and results of prenatal diagnosis methods.

**Prenatal diagnosis method**	**Total**	**Euploidies**	**Balanced translocation**	**Aneuploidies**					**Positive rate**
				**Common aneuploidies (T21/T18/T13)**	**Sex chromosome aneuploidies**	**Segmental aneuploidies**			
						**P**	**LP**	**VUS**	
Karyotyping	63	60	1		2				4.76%
CMA/CNV-seq	72	61			1^a^	4	2	4	15.28%

*CMA, chromosomal microarray analysis; CNV-seq, copy number variation sequencing; P, pathogenic; LP, likely pathogenic; VUS, variant of uncertain significance. ^*a*^The CNV-seq and QF-PCR (quantitative fluorescence polymerase chain reaction) results of this case were inconsistent. CNV-seq did not indicate abnormalities, while QF-PCR indicated abnormalities in the sex chromosome sites. Due to the doubtful results, additional FISH (fluorescence *in situ* hybridization) analysis was added. FISH results suggested mosaic XX/XY.*

Regarding the pregnancy outcomes of fetuses with chromosomal abnormalities indicated by prenatal diagnosis, nine cases chose to continue the pregnancy, whereas five chose to terminate it (see Table 2).

## Discussion

Due to a low fetal fraction and other factors, NIPS detection failure is inevitable. In a recent meta-analysis on NIPS, Gil et al. (2017) analyzed failure rates ranging from 0.0 to 12.2%. Further details were provided by 11 of the 35 studies, showing that the detection failure rate of the low fetal fraction ranged from 0.1 to 6.1%. In this study, the overall test failure rate and the rate of test failure due to low fetal fraction were 0.32 and 0.25%, respectively, which is consistent with other reports.

Common reasons for the failure of NIPS include low fetal fraction, high or low total cell-free DNA concentration in plasma, low DNA library concentration, sequencing failure, experimental testing procedures, or failure to meet other quality control standards (Qiao et al., 2019). The main reason is the low fetal fraction, which is consistent with the results obtained in this study. The cause of the low fetal fraction may be related to gestational age, maternal weight, maternal age, ethnicity, assisted conception, or heparin use, among others (Rijnders et al., 2003; Hudecova et al., 2014; Hui, 2016; Revello et al., 2016; Ma et al., 2018). In this study, the gestational age of the low fetal fraction group was 17.40 ± 2.27 W and the BMI was 25.66 ± 3.67 kg/m^2^, which were significantly different from those of the sequencing failure group and the DNA concentration outside of laboratory quality control group (all *P* < 0.05, see Table 1). This shows that a low gestational age and high BMI are more likely to lead to a low fetal fraction, thus causing test failure, which is consistent with previous studies.

The invasive prenatal diagnosis method used in this study was amniocentesis. Of the 378 cases with follow-up results who failed the test, only 135 (34.26%) pregnant women chose further invasive prenatal diagnosis. Some pregnant women may resist invasive prenatal diagnosis due to other reasons, such as low risk of serological screening in the second trimester and no abnormalities indicated by ultrasound examination. Additionally, amniocentesis and chorionic villus sampling (CVS) may bring a certain degree of risk of abortion or other surgical complications (Tabor and Alfirevic, 2010; Beta et al., 2019). The rate of fetal loss during pregnancy for amniocentesis and CVS is approximately 1 in 1,000 and 1 in 500, respectively (Akolekar et al., 2015; Abel and Alagh, 2020). There were no cases of fetal loss or other puncture complications due to invasive methods in this study. On the other hand, prenatal diagnostic tests performed after invasive surgery have a high success rate, enabling pregnant women to make better decisions about pregnancy management based on the results of subsequent invasive prenatal diagnosis. Literatures have shown that although the success rate of conventional karyotype analysis in abortion or stillbirth samples was only about 70–80% (Sahlin et al., 2014; Pauta et al., 2018; Martinez-Portilla et al., 2019), the overall success rate in testing prenatal samples (amniotic fluid, umbilical cord blood, or villus tissue) can be as high as 98–99% (Ocak et al., 2014; Tao et al., 2019; Wang et al., 2020; Zhuang et al., 2021) and the overall success rate of high-resolution molecular genetics technology (CMA, CNV-seq, QF-PCR, and BACs-on-Beads assay, etc.) was about 95–100% (Sahlin et al., 2014; Hu et al., 2017; Pauta et al., 2018, 2020; Wang et al., 2018, 2020; Martinez-Portilla et al., 2019; Tao et al., 2019; Zhuang et al., 2021), and almost not affected by sample types. Therefore, it is a less risky option for a pregnant woman to undergo a prenatal diagnostic test after NIPS failure than to not undergo further prenatal diagnostic tests to determine whether the fetus has chromosomal abnormalities.

Among the NIPS failures in this study, six cases were found to have developmental abnormalities after delivery (Table 4). Although no abnormality was found in prenatal karyotype examination of case S1 and case S2, the possibility of microdeletion, microduplication, or other copy number variation could not be ruled out because karyotype analysis could only identify chromosomal abnormality larger than 5–10 Mb. Even so, their parents refused to perform other high-resolution examination methods. The condition of case S1 improved after rehabilitation treatment, and subsequent surgical treatment was planned for both case S2 and case S5. Case S3 was diagnosed as DiGeorge syndrome in prenatal testing. Most of the children with DiGeorge syndrome had growth and development retardation and language retardation in the early stage and were often accompanied by congenital malformations in various parts of the body. The mother of the child still chose to continue pregnancy after being informed of the possible abnormality of the child. The whole exome sequencing was performed on Case S4 due to the postpartum comprehensive developmental retardation, and the results suggested that the child had a single-gene disease (Mental retardation, autosomal dominant 18), which was confirmed to be a *de novo* mutation. The chromosomal abnormalities in cases S3 and S4 would be missed by conventional karyotype analysis.

Different studies have pointed out that NIPS test failures may be associated with increased risk of trisomy 18, trisomy 13, monomer X, and triploid (Pergament et al., 2014; Norton et al., 2015; Palomaki et al., 2015; Revello et al., 2016; Suzumori et al., 2016; Samura and Okamoto, 2020). In this study, 15 chromosomal abnormalities were detected from prenatal or postnatal testing, of which copy number variation accounted for 66.67% (*n* = 10), sex chromosome abnormal mosaicism for 13.33% (*n* = 2), chromosome aneuploidy for 6.67% (*n* = 1), balanced translocation for 6.67% (*n* = 1), and SNV for 6.67% (*n* = 1). The type of fetal chromosomal abnormality that failed NIPS in this study was mainly copy number variation, which was different from previous research results. This may be caused by inconsistencies within the population and the diagnostic methods used in our study and previous studies. Table 6 presents a summary of chromosomal abnormalities in NIPS failure cases from different studies in recent years (Pergament et al., 2014; Norton et al., 2015; Revello et al., 2016; McKanna et al., 2019; Hancock et al., 2020; Lopes et al., 2020; Guy et al., 2021). For example, Norton et al. (2015) detected six common aneuploidies (trisomy 21, trisomy 18, and trisomy 13), four triploidies, one trisomy 16 mosaic, one deletion 11p, and one structurally abnormal chromosome. Their study of triploid and chromosome aneuploidy (including mosaicism) accounted for 2.25% (11/488), which was significantly higher than the 0.79% (3/378) observed in our study. This may be associated with the lower gestational age of their study population (average 12.5 W) than in our study (average 17.78 W). Nearly half of all early pregnancy losses are due to chromosomal abnormalities, with triploidy being the main cause (Stephenson et al., 2002). On the other hand, most of the cases with abnormal pregnancy outcomes (induced labor, miscarriage, and abnormal postnatal development), which were likely at high risk for triploidy and aneuploidy, did not undergo chromosome examinations in our study. The overall chromosomal abnormality rate in the study by Norton et al. (2015) was 2.7%, which was lower than the 3.97% (15/378) in our study. This may be due to the different prenatal diagnostic methods used in the two studies. The prenatal diagnostic method used in almost all of the studies in Table 6 was karyotype analysis. We used karyotype analysis in 46.7% of the cases. The other diagnostic method in our study was CMA/CNV-seq (53.3%). The resolution of CMA/CNV-seq is higher than that of karyotype analysis, which improves the detection rate of chromosomal abnormalities (Hay et al., 2018; Wang et al., 2018). In those studies shown in Table 6, the overall older maternal age may also be the reason for the high incidence of aneuploidy. The risk of chromosomal structural abnormalities does not increase with maternal age; on the contrary, the probability of aneuploidy abnormalities increases with maternal age (Committee on Practice Bulletins-Obstetrics, Committee on Genetics, and The Society for Maternal–Fetal Medicine, 2016a).

**TABLE 6:** Summary of chromosomal abnormalities in cases of NIPS test failure in different studies.

**Study (author, year)**	**Test failures (n)**	**Triploidy (n)**	**Aneuploidy (n)**	**Chromosomal structural abnormality**	**The rate of chromosomal abnormality (%)**	**Average gestational age (weeks)**	**Maternal age (years)**	**Invasive prenatal diagnosis test**
Norton et al., 2015	488	4	7	2	2.7	12.5	31 (range between 18 and 48)	Not available
Revello et al., 2016	308		8		2.6	Range between 10.0 and 14.0	36.3 (range between 33.2 and 39.3)	Karyotyping
Pergament et al., 2014	85		19		22.4^a^	14.6	30.3	Karyotyping or FISH
Guy et al., 2021	32		6		18.8^a^	12.0 (range between 11.0 and 14.0)	34.6 (range between 31.1 and 38.1)	Karyotyping
Hancock et al., 2020	316		24		7.6	About 12.0(range between 10.0 and 41.5)	About 35.0 (ranged between 18 and 45)	Not available
McKanna et al., 2019	1148	21	25	2	4.2	12.3	34.0 (range between 17 and 48)	Karyotyping
Lopes et al., 2020	22	1	1		9.1^a^	13.7	32.3	Not available

*^*a*^The overall number of test failures in the study was small, and the proportion of chromosomal abnormalities may be biased by the data.*

According to the follow-up results, the incidence of chromosomal abnormalities in NIPS test failure cases could reach 3.97%. This was higher than that of chromosomal abnormalities in the common population (1.8%), which was mentioned in previous reports (Evans et al., 2016). These results indicate that pregnant women who fail NIPS need to undergo adequate genetic counseling and focus on invasive prenatal diagnosis. Meanwhile, the positive chromosomal abnormality detection rate in the CMA/CNV-seq group was higher than that in the karyotyping group in our study. A total of 73.33% (11/15) of chromosomal abnormalities in our study were indicated as CNV or SNV. This incidence rate is sufficient to indicate that the selection of invasive prenatal diagnosis methods cannot be limited to karyotyping; rather, high-resolution detection methods are required.

## Data Availability Statement

The data analyzed in this study is subject to the following licenses/restrictions: The datasets for this article are not publicly available because of privacy concerns. Requests to access these datasets should be directed to QZ, zhuqian_2009@163.com.

## Ethics Statement

The studies involving human participants were reviewed and approved by the Institutional Ethics Committee of Sichuan University. The patients/participants provided their written informed consent to participate in this study.

## Author Contributions

SL, QZ, and HL designed the research. SL, TB, XJ, CD, TX, XW, JC, LX, YYL, and YL carried out the experiments. SL, JL, QZ, and HL analyzed the experimental results and sequencing data. QFZ, XJ, YYL, and LX participated in the follow-up. SL wrote the main manuscript. QZ supervised the project and offered valuable suggestions for the revision of the manuscript. All authors participated in the discussion of the results and approved the final manuscript.

## Conflict of Interest

The authors declare that the research was conducted in the absence of any commercial or financial relationships that could be construed as a potential conflict of interest.

## Footnotes

1 http://dgv.tcag.ca/

2 https://decipher.sanger.ac.uk/

3 http://omim.org/

4 https://www.ncbi.nlm.nih.gov/pubmed

5 http://www.gpmedical.com.cn
